# Vitamin D Binding Protein Is Not Involved in Vitamin D Deficiency in Patients with Chronic Kidney Disease

**DOI:** 10.1155/2015/492365

**Published:** 2015-05-04

**Authors:** Marta Kalousova, Sylvie Dusilova-Sulkova, Oskar Zakiyanov, Milada Kostirova, Roman Safranek, Vladimir Tesar, Tomas Zima

**Affiliations:** ^1^Institute of Medical Biochemistry and Laboratory Diagnostics, First Faculty of Medicine, Charles University in Prague and General University Hospital in Prague, U Nemocnice 499/2, 128 01 Prague, Czech Republic; ^2^Haemodialysis Centre, University Hospital Hradec Kralove, Sokolská 581, 500 05 Hradec Kralove, Czech Republic; ^3^Department of Nephrology, First Faculty of Medicine, Charles University in Prague and General University Hospital in Prague, U Nemocnice 499/2, 128 08 Prague, Czech Republic

## Abstract

*Objective*. This study was designed to evaluate vitamin D status with separate determination of 25-OH D_2_ and 25-OH D_3_ and its relationship to vitamin D binding protein (VDBP) in patients with chronic kidney disease (CKD) and long-term haemodialysis patients (HD). *Methods*. 45 CKD patients, 103 HD patients, and 25 controls (C) were included. Plasma vitamin D concentrations were determined using chromatography and VDBP in serum and urine in CKD using enzyme immunoassay. *Results*. Plasma vitamin D levels were lower in CKD (30.16 ± 16.74 ng/mL) and HD (18.85 ± 15.85 ng/mL) versus C (48.72 ± 18.35 ng/mL), *P* < 0.0001. 25-OH D_3_ was the dominant form of vitamin D. Serum VDBP was higher in CKD (273.2 ± 93.8 ug/mL) versus C (222 ± 87.6 ug/mL) and HD (213.8 ± 70.9 ug/mL), *P* = 0.0003. Vitamin D/VDBP ratio was the highest in C and the lowest in HD; however, there was no correlation between vitamin D and VDBP. Urinary concentration of VDBP in CKD (0.25 ± 0.13 ug/mL) correlated with proteinuria (*r* = 0.43, *P* = 0.003). *Conclusions*. Plasma levels of vitamin D are decreased in CKD patients and especially in HD patients. 25-OH D_3_ was the major form of vitamin D. Despite urinary losses of VDBP, CKD patients had higher serum VDBP concentrations, indicating compensatory enhanced production. Vitamin D binding protein is not involved in vitamin D deficiency.

## 1. Introduction

Vitamin D plays important physiological roles in maintaining calcium and phosphate homeostasis but also in many other biological processes. There is growing evidence that low vitamin D status is associated with several diseases including not only osteoporosis and osteomalacia but also cardiovascular disease, diabetes mellitus, multiple sclerosis, rheumatoid arthritis, and other autoimmune conditions and several cancer types [1, 2]. In general, regulating the transcription of many genes through their binding to nuclear vitamin D receptor (VDR), active vitamin D serves as antiproliferative and prodifferentiating factor [1, 2].

Vitamin D may be produced endogenously in the skin (ultraviolet irradiation converting 7-dehydrocholesterol to cholecalciferol, i.e., vitamin D_3_) or obtained from food or supplements (mostly ergocalciferol, i.e., vitamin D_2_, but also D_3_, e.g., from fish sources). To become biologically active, both ergocalciferol and cholecalciferol must be double-hydroxylated. The product of the first hydroxylation in the liver, 25-hydroxyvitamin D (calcidiol, 25-OH D), is the major circulating vitamin D metabolite. Calcidiol is then converted by the second (1*α*) hydroxylation to calcitriol, that is, dihydroxylated active form of vitamin D (1, 25-OH D). For production of circulating calcitriol, renal 1*α*-hydroxylase is responsible, but also several nonrenal tissues and cell lines express their own 1*α*-hydroxylase activity [1–4]. As the liver hydroxylation is neither regulated nor rate limited, 25-OH vitamin D well represents vitamin D status in the body. Serum 25-OH D levels > 75 nmol/L (30 ng/mL) indicate sufficient vitamin D stores [1, 4].

Many CKD and mainly HD patients have low serum or plasma vitamin D concentrations [5–11]. Several explanations have been suggested: low solar radiation exposure, disturbed conversion of vitamin D precursor in the skin, low food intake of vitamin D, and loss of vitamin D binding protein due to proteinuria and accelerated vitamin D catabolism [4, 12, 13]. However, the definite role of these possible causes is not clear.

Vitamin D binding protein (VDBP) is a 58 kDa circulating alpha globulin produced primarily by the liver, binding the majority (>85%) of circulating 25-OH vitamin D [14]. It is a highly polymorphic single chain serum glycoprotein ensuring that circulating vitamin D is delivered to target tissues [15]. In principle, there are two main roles of VDBP in vitamin D physiology: enlargement of biological half-life of vitamin D (as binding protects vitamin D from biodegradation) and limiting its access to target tissues. Moreover, VDBP maintains plasma vitamin D levels through reabsorption in the kidneys [16]. The complex of VDBP with 25-OH vitamin D is filtered in the glomerulus, which is followed by receptor mediated reuptake at the brush border of tubular epithelial cells [17] involving megalin [18] and cubilin [19]. In addition to its vitamin D binding properties, there are additional actions attributed to VDBP including binding of extracellular actin and transport of fatty acids. VDBP also appears to protect the complement C5a from proteolytic degradation, enhancing its action as chemotactic protein [20]. A deglycosylated form of VDBP, VDBP-macrophage activating factor, is able to promote activation of macrophages and osteoclasts, and even native VDBP may have effect on osteoclasts [21].

There are several methodological approaches for vitamin D determination in serum or plasma. They can be grouped into immunochemical methods (based on radioactive, enzymatic, or chemiluminescence detection), chromatographic methods (HPLC: high-performance liquid chromatography), and mass spectrometry. Immunochemical methods may vary due to differential detection of D_3_ and D_2_ molecules (conventional analytical measurement of serum or plasma 25-OH D level reflects the sum of 25-OH D_3_ plus 25-OH D_2_), interference by detection using polyclonal antibodies, and nonspecific detection of other vitamin D metabolites including degradation products [22, 23]. In addition, incomplete release of vitamin D from VDBP has been identified as a potential source of variability for both manual and automated immunoassays [24]. Another analytical approach, HPLC (high-performance liquid chromatography), allows not only precise assessment of vitamin D level but also simultaneous measurement of 25-OH D_2_ and 25-OH D_3_ vitamins separately, informing thus about the source of vitamin D.

Therefore, in the current study, the aim was to measure vitamin D concentration in plasma using HPLC method with separate detection of vitamin D_2_ and vitamin D_3_ and to assess VDBP levels in serum in patients with CKD and HD patients and healthy subjects for comparison. In addition, urine levels of VDBP in CKD patients were also assessed to allow a more complex view of vitamin D status.

## 2. Materials and Methods

This is a cross-sectional study that includes 173 subjects with CKD, long-term HD and healthy controls. All patients were in stable clinical status at the time of the study, without signs of acute infection. Vitamin D supplementation (apart from dihydroxylated form of vitamin D3) was not prescribed in any HD or CKD patients at the time of study, and controls did not take any special alimentary supplements. The study was approved by the Ethical Committee and all patients have given written informed consent prior to entering the study.

### 2.1. Study Groups

#### 2.1.1. CKD Patients

Forty-five patients (27 male and 18 female, mean age 60 ± 17 years) with CKD were included. Their median creatinine clearance was 0.39 mL/s/1.73 m^2^ (IQR 0.22–0.70 mL/s/1.73 m^2^). Urinary protein concentration was 0.22 g/L (median; IQR 0.09–1.10 g/L) and daily protein losses varied from 0.04 g to 13.78 g (median 0.48 g/24 hours). Duration of their follow-up for renal disease was 84 months (IQR 24–120 months). Causes of their renal disease were diabetic nephropathy in 2 patients, hypertensive nephropathy in 14 cases, chronic tubulointerstitial nephritis in 5 patients, chronic glomerulonephritis in 12 cases, polycystic kidney disease in 6 patients, and multifactorial or unknown in 6 cases. The majority of patients (42 cases) had hypertension and were treated with moderate doses of antihypertensive drugs. Cardiovascular disease was known in eight patients. Twelve patients had diabetes mellitus and were treated with insulin or peroral antidiabetics. Twenty-eight patients had dyslipidaemia and were treated with statins. Basic laboratory characteristics of CKD patients are given in Table 1.

#### 2.1.2. HD Patients

One hundred and three patients with end-stage renal disease treated on long-term HD (63 male and 40 female, mean age 60 ± 14 years) were included in the study. Their primary renal diagnoses were as follows: diabetic nephropathy (*N* = 26), vascular nephropathy (*N* = 5), tubulointerstitial nephritis (*N* = 22), chronic glomerulonephritis (*N* = 21), polycystic kidney disease (*N* = 14), and other or unknown diagnoses (*N* = 15). Their residual diuresis ranged from anuria to 2500 mL. The majority of patients were dialyzed three times weekly for 4–4.5 hours using conventional bicarbonate-buffered dialysate. The majority of patients (97 cases in total) had hypertension and were treated with moderate doses of antihypertensive drugs. Sixty-one patients had dyslipidaemia. Cardiovascular disease was known in 34 cases. Forty-one patients had diabetes mellitus and were treated with insulin or peroral antidiabetics. Patients with secondary hyperparathyroidism were treated by synthetic calcitriol (i.e., dihydroxylated form of vitamin D_3_) or paricalcitol (synthetic analogue of vitamin D_2_). None of patients received any native vitamin D or its 25-hydroxylated metabolite. Basic laboratory characteristics of HD patients are provided in Table 1.

#### 2.1.3. Control Group

The control group (C) consisted of 25 healthy adults (15 male and 10 female, mean age 49 ± 10 years). Basic laboratory characteristics of controls are shown in Table 1.

### 2.2. Samples

In HD patients, blood was collected from inserted dialysis needle into the arteriovenous fistula before starting HD session and prior to heparin administration. In other subjects, blood was collected after overnight fasting by puncturing the cubital vein with simultaneous blood collection for routine control examinations. Routine laboratory parameters were measured in fresh samples according to institutional standards. For vitamin D determination and VDBP assessment, blood was centrifuged for 10 minutes at 1450 g, and serum and plasma were frozen at −80°C until analysis. Additionally in CKD patients, a 24-hour urine sample was collected, frozen at −80°C, and used for analysis.

### 2.3. Laboratory Analyses

Vitamin D (its hydroxylated form, 25-OH D) in plasma was assessed with Chromsystems reagent kit (http://www.chromsystems.de) using high-performance liquid chromatography. This assay allows simultaneous determination of 25-hydroxycholecalciferol (25-OH D_3_) and 25-hydroxyergocalciferol (25-OH D_2_). Samples were treated according to the manufacturer's protocol and HPLC was performed with isocratic system with UV detection (HPLC apparatus ECOM; ECOM, http://www.ecom.cz/).

Vitamin D binding protein (VDBP) in serum and urine was assessed by standard ELISA (enzyme linked immunosorbent assay) Quantikine, RD Systems (Minneapolis, MN, USA), according to the manufacturer's protocol.

For parathyroid hormone (PTH) determination, second generation test was used (iPTH), ECLIA (Electrochemiluminescence, Modular, Roche, Germany). Other laboratory parameters were measured with standard methods.

### 2.4. Statistical Analysis

Statistical software GraphPad Prism 6 (GraphPad Software, San Diego, CA, USA) was used for statistical evaluation. Results are expressed as mean ± standard deviation and in case of high interindividual variability also as medians (interquartile ranges). Vitamin D_3_/VDBP and total vitamin D/VDBP ratio was calculated and evaluated. Comparison among groups was done with one-way ANOVA (analysis of variance) test followed by Tukey's multiple comparison test. Correlations were tested using Pearson and Spearman correlation coefficients. All tests were two-sided and results were considered statistically significant for *P* < 0.05.

## 3. Results

### 3.1. Vitamin D Status

Plasma vitamin D levels were significantly lower in CKD (30.16 ± 16.74 ng/mL) and HD (18.85 ± 15.85 ng/mL) patients versus (48.72 ± 18.35 ng/mL) in controls, *P* < 0.0001 (Table 2). In particular, low levels were in HD patients and more than 75% of HD patients were vitamin D deficient. 25-OH D_3_ was the dominant form of vitamin D (Table 2) and in most subjects serum 25-OH D_2_ was not detectable. This was the case for all control subjects and CKD patients. Also in HD patients, serum 25-OH D_2_ was detectable only in few patients, but these concentrations were low. Overall, the total 25-OH D levels were represented mostly by 25-OH D_3_. Vitamin D was not detectable in urine in CKD patients.

Vitamin D_3_ significantly correlated with haemoglobin in CKD patients but not in HD patients and controls. In HD patients, but not in CKD patients and controls, plasma D_3_ positively correlated with serum calcium. In CKD patients, vitamin D_3_ correlated with proteinuria, protein losses, and diuresis but not with serum creatinine and creatinine clearance. No other correlations between plasma vitamin D and investigated laboratory parameters were found in HD and CKD patients, as well as in control group. Significant correlations are summarized in Table 3.

### 3.2. Vitamin D Binding Protein

Serum VDBP levels in CKD patients were significantly higher compared to controls as well as to HD patients (Table 2). There was no association between VDBP and vitamin D_3_ in any studied group of subjects. VDBP negatively correlated with age in CKD patients which was not proven either in HD patients or in controls. VDBP did not correlate either with serum creatinine or with creatinine clearance in these patients and was not related to serum albumin, calcium, phosphate, PTH, C-reactive protein, or body mass index (BMI). Only in HD patients, VDBP correlated slightly positively with serum albumin and slightly negatively with BMI.

Vitamin D_3_/VDBP ratio and total vitamin D/VDBP ratio significantly differed among studied groups with the highest levels in controls and the lowest levels in HD patients (Table 2) but did not correlate with basic laboratory parameters in any of the studied groups apart from haemoglobin in CKD patients.

Urinary concentration of VDBP in CKD patients (0.25 ± 0.13 ug/mL) correlated with proteinuria. Moreover, urinary VDBP positively correlated with serum creatinine in CKD patients and similar relationship was found between creatinine clearance and VDBP in urine. No association between serum and urinary VDBP was found. Significant correlations are summarized in Table 3.

Taken together, the majority of patients with chronic kidney diseases are 25-OH vitamin D deficient. Vitamin D is represented mainly by 25-OH vitamin D_3_ (25-hydroxycholecalciferol). Serum VDBP is increased in CKD patients and is measurable also in urine. Vitamin D/VDBP is the highest in healthy subjects and the lowest in HD patients.

## 4. Discussion

Examining a cohort of CKD and HD patients and a control group in a cross-sectional design, we assessed vitamin D status by separate determination of both 25-hydroxylated vitamin D metabolites (hydroxyergocalciferol, i.e., 25-OH D_2_, and hydroxycholecalciferol, i.e., 25-OH D_3_) in plasma. This allowed us to recognize the probable source of vitamin D for given subjects. Concomitant measurement of VDBP in serum and also in urine in CKD patients allowed us to evaluate the possible role of urinary VDBP losses in vitamin D status.

We found significantly lower 25-OH D plasma levels in CKD patients compared to controls. In line with previous studies, the majority of CKD patients were vitamin D deficient. In particular, low levels were found in HD patients, which is also in agreement with published data [5, 7–9]. Contrary to previous published studies, we measured separately 25-OH D_2_ and 25-OH D_3_ which provided us with information about the source of vitamin D in these subjects. Surprisingly, 25-OH D_2_ was not detectable in plasma in the great majority of studied subjects. None of controls and CKD patients and only few HD patients exhibited detectable 25-OH D_2_. Undetectable 25-OH D_2_ found in plasma in all control subjects and also in the majority of renal patients points out very low intake of vitamin D from plant sources. Further studies are necessary to confirm if this observation can be generalized for our country or even for other geographical and socioeconomic areas.

Undetectable 25-OH D_2_ and numerical values of 25-OH D_3_ plasma concentration in our subjects indicate either solar skin irradiation and/or food containing vitamin D_3_ (e.g., fish) as a vitamin D source. However, low 25-OH D_3_ in CKD patients not yet on dialysis and not with limited life style indirectly shows that causes other than low sun exposure and/or disturbed conversion of skin precursor play a role in their vitamin D deficiency. Metabolic changes and their consequences that accompany CKD and end-stage renal disease may influence 25-OH D plasma levels in CKD patients and mainly in HD patients. For example, reduced hepatic synthesis of calcidiol in uraemia was described recently [25]. However, very high PTH level, which is considered as the main factor responsible for disturbed hepatic hydroxylation, was not the case in our patients.

Serum concentrations of VDBP were similar in healthy controls and HD patients. However, we observed significantly increased levels of VDBP in CKD patients compared to controls as well as to HD patients, which is in contrast to other studies [26, 27]. This increase was observed despite the urinary VDBP losses which were independent of serum VDBP levels. Urine concentrations of VDBP were as low as several tenths of ug per mL, but total daily amount was not negligible and, however, still did not result in serum VDBP decrease. We might speculate that in vitamin D deficiency VDBP would increase. However, this would explain just the serum VDBP increase in CKD patients but not normal VDBP levels in serum in HD patients among whom many do not experience any urinary VDBP losses due to anuria. However, the interpretation of elevated VDBP should be more complex, as an association between high VDBP and risk of several cancers has been described [28]. Regardless of the mechanism leading to VDBP elevation, it is obvious that VDBP losses in urine in our patients were not associated either with vitamin D status or with serum VDBP levels similarly as in one previous study [26]. VDBP in urine was inversely related to creatinine clearance and urinary DBP correlated with proteinuria which is in line with the above-mentioned study [26], where additional urinary VDBP excretion responded to antiproteinuric treatment and was higher than that in healthy subjects. This is consistent with previously published hypothesis that urinary DBP is a marker of renal interstitial inflammation and fibrosis [29]. The occupancy of circulating VDBP by vitamin D metabolites is generally lower than 5% [14]. When calculating the 25-OH D/VDBP ratio (resp., 25-OH D_3_/VDBP ratio), the main result was that this ratio is much higher in healthy controls than in CKD and HD patients. This further supports the finding that vitamin D deficiency observed in CKD and HD patients was not related to VDBP serum concentration. Also the lack of association between serum VDBP and 25-OH D levels supports the conclusion that VDBP has little effect on concentrations of vitamin D metabolites.

However, we focused only on total vitamin D serum level and we did not consider its free or so-called bioavailable fraction. According to free hormone hypothesis, only unbound fraction is biologically active [30]. With respect to vitamin D physiology, a recent study found that lower VDBP resulted in higher vitamin D bioavailability [31]. Higher bioavailability may lead to higher biological effect but also to higher biodegradation, that is, shorter half-life. Thus, the definite answer if the assessment of bioavailable fraction will bring superior information compared to total vitamin D serum/plasma level is not known at present.

Besides the slightly positive link between 25-OH D_3_ and serum calcium in HD patients, vitamin D status did not correlate with any measured parameter of bone and mineral metabolism. In particular, no correlation between 25-OH D_3_ and iPTH was found. Several papers report on contribution of low vitamin D status to secondary hyperparathyroidism [32]. In our patients, vitamin D status was generally low but the lack of the direct association between PTH and 25-D concentrations is not surprising as 25-OH D_3_ does not represent an active vitamin D form. Active form, dihydroxylated vitamin D (calcitriol), is synthetized by kidneys but also by many extrarenal tissues and cell lines. Low serum calcitriol belongs to main stimulators for PTH production and triggers the initial as well as advanced forms of secondary hyperparathyroidism [33, 34]. We did not measure calcitriol serum concentration and thus we cannot discuss this topic in the view of our data. However, according to recent data, low calcitriol production in CKD and HD patients is related not only to kidney dysfunction but also to low substrate, that is, low 25-OH D levels [4]. Based on this, it is important to assess vitamin D status, which was the main target in our study. The positive association of plasma vitamin D_3_ concentration with proteinuria was surprising and requires further investigation.

In CKD patients, but not in HD patients and controls, serum VDBP levels inversely correlated with age. The age dependency of vitamin D binding protein was described also by others [35, 36]. We did not analyse this relationship, but it is not likely to be associated with age-dependent decrease of renal function, as there was no relationship between serum creatinine and VDBP. Contrary to CKD subjects, in HD patients, serum VDBP positively correlated with serum albumin and inversely with BMI, indicating possible nutritional association.

The present study has several limitations. The age of our groups of patients was a little bit different, which theoretically might influence VDBP concentration, as we found a correlation between age and VDBP but only in CKD patients. Another issue which complicates the interpretation of our findings is well-known genetic variability in VDBP [14]. Vitamin D was assessed in plasma and VDBP in serum which was done due to the availability of material and according to manufacturers, and both methods were designed for both materials. Lastly, no vitamin D nutritional supplementation was prescribed in our patients, but the possibility of vitamin D intake cannot be rigorously excluded. Although some patients were treated with active vitamin D or paricalcitol, it was demonstrated recently that they do not affect 25-OH D levels in blood [10].

## 5. Conclusion

In CKD and mainly HD patients not administered vitamin D supplementation, low vitamin D status was found, with no detectable 25-OH D_2_ in most cases. Thus, 25-OH D_3_ was the major form of vitamin D. Despite VDBP urinary losses in nondialysis CKD patients, serum VDBP was increased in these subjects compared to healthy controls, indicating VDBP losses are not responsible for 25-OH D deficiency.

## Figures and Tables

**Table 1:** Characteristics of study subjects.

	Healthy controls (C) *N* = 25	Chronic kidney disease (CKD) *N* = 45	Long-term haemodialysis (HD) *N* = 103	Significance *P*
Body mass index (kg/m^2^)	26.13 ± 4.28	26.84 ± 5.18	27.07 ± 5.34	n.s.

S-Creatinine (umol/L)	91 ± 12	292 ± 148	777 ± 219	*P* < 0.0001 C versus CKD^***^ C versus HD^****^ CKD versus HD^****^

Haemoglobin (g/L)	143 ± 9	120 ± 14	112 ± 11	*P* < 0.0001 C versus CKD^****^ C versus HD^****^ CKD versus HD^***^

Albumin (g/L)	47.3 ± 3.4	41.4 ± 5.0	40.9 ± 3.3	*P* < 0.0001 C versus CKD^****^ C versus HD^****^ CKD versus HD n.s.

Calcium (mmol/L)	2.32 ± 0.11	2.28 ± 0.25	2.16 ± 0.18	*P* < 0.0001 C versus CKD n.s. C versus HD^***^ CKD versus HD^**^

Phosphate (mmol/L)	1.13 ± 0.20	1.19 ± 0.30	1.95 ± 0.57	*P* < 0.0001 C versus CKD n.s. C versus HD^****^ CKD versus HD^****^

Parathyroid hormone (pmol/L)	4.21 ± 2.26 3.73 (3.02–4.75)	14.03 ± 14.43 9.60 (5.15–16.73)	34.57 ± 35.81 23.97 (14.60–41.05)	*P* < 0.0001 C versus CKD n.s. C versus HD^****^ CKD versus. HD^***^

C-reactive protein (mg/L)	6.1 ± 3.0 5.6 (3.8–7.1)	7.5 ± 8.2 5.0 (2.4–7.2)	6.9 ± 6.9 5.5 (2.0–9.0)	n.s.

Proteinuria (g/L)	Not assessed (dipstick negative)	0.22 (0.09–1.10)	Not assessed	Not evaluated

Results are expressed as mean ± standard deviation and in case of high interindividual variability also as medians (interquartile ranges).

Comparison: one-way ANOVA and Tukey's multiple comparison test.

^****^ *P* < 0.0001, ^***^ *P* < 0.001, ^**^ *P* < 0.01.

**Table 2:** Vitamin D and vitamin D binding protein in study groups.

	Healthy controls (C) *N* = 25	Chronic kidney disease (CKD) *N* = 45	Long-term haemodialysis (HD) *N* = 103	Significance *P*
25-OH vitamin D_3_ in plasma (ng/mL)	48.72 ± 18.35 47.10 (37.10–65.40)	30.16 ± 16.74 24.65 (19.45–38.50)	18.09 ± 15.64 13.70 (9.98–22.65)	*P* < 0.0001 C versus CKD^****^ C versus HD^****^ CKD versus HD^***^

25-OH vitamin D_2_ in plasma (ng/mL)	Not detected	Not detected	Detected in 8 patients: 9.67 ± 7.80 8.08 (1.83–15.10)	Not evaluated

Total 25-OH vitamin D (D_2_ + D_3_) in plasma (ng/mL)	48.72 ± 18.35 47.10 (37.10–65.40)	30.16 ± 16.74 24.65 (19.45–38.50)	18.85 ± 15.85 14.45 (10.33–23.65)	*P* < 0.0001 C versus CKD^****^ C versus HD^****^ CKD versus HD^***^

VDBP in serum (ug/mL)	222.0 ± 87.7 209.0 (163.0–269.2)	273.2 ± 93.8 268.0 (217.4–327.4)	213.8 ± 70.9 206.6 (161.5–252.3)	*P* = 0.0003 C versus CKD^*^ C versus HD n.s. CKD versus HD^***^

VDBP in urine (ug/mL)	Not assessed	0.25 ± 0.13 0.33 (0.12–0.35)	Not assessed	Not evaluated

25-OH vitamin D_3_/VDBP ratio (×10^−6^)	246.56 ± 112.44 242.54 (153.02–275.30)	129.72 ± 93.57 97.93 (72.34–191.86)	90.97 ± 74.97 70.84 (40.91–116.39)	*P* < 0.0001 C versus CKD^****^ C versus HD^****^ CKD versus HD^*^

Total 25-OH vitamin D (D_2_ + D_3_)/VDBP ratio (×10^−6^)	246.56 ± 112.44 242.54 (153.02–275.30)	129.72 ± 93.57 97.93 (72.34–191.86)	95.26 ± 77.16 70.84 (42.23–120.172)	*P* < 0.0001 C versus CKD^****^ C versus HD^****^ CKD versus HD n.s.

Results are expressed as mean ± standard deviation and in case of high interindividual variability also as medians (interquartile ranges).

Comparison: one-way ANOVA and Tukey's multiple comparison test.

^****^ *P* < 0.0001, ^***^ *P* < 0.001, and ^*^ *P* < 0.05.

VDBP: vitamin D binding protein.

**Table 3:** Overview of significant correlations of vitamin D and vitamin D binding protein in the study groups.

Chronic kidney disease patients			
25-OH vitamin D_3_	VDBP in serum	25-OH vitamin D_3_/VDBP ratio	VDBP in urine

Haemoglobin (*r* = 0.40, *P* = 0.006) Proteinuria (*r* = 0.34, *P* = 0.02) Protein losses (*r* = 0.43, *P* = 0.003) Diuresis (*r* = 0.40, *P* = 0.006)	Age (*r* = −0.34, *P* = 0.02)	Haemoglobin (*r* = 0.31, *P* = 0.04)	Proteinuria (*r* = 0.43, *P* = 0.003) Protein losses (*r* = 0.39, *P* = 0.008) Serum creatinine (*r* = 0.47, *P* = 0.001) Creatinine clearance (*r* = −0.41, *P* = 0.006)

Long-term haemodialysis patients			
25-OH vitamin D_3_	VDBP in serum	25-OH vitamin D_3_/VDBP ratio	

Calcium (*r* = 0.22, *P* = 0.03)	Albumin (*r* = 0.22, *P* = 0.03) BMI (*r* = −0.22, *P* = 0.03)		

Controls			
25-OH vitamin D_3_	VDBP in serum	25-OH vitamin D_3_/VDBP ratio	

No significant correlation.			

VDBP: vitamin D binding protein.
